# Unwinding a spiral of cellulose nanocrystals for stimuli-responsive stretchable optics

**DOI:** 10.1038/s41467-019-08351-6

**Published:** 2019-01-31

**Authors:** Osamu Kose, Andy Tran, Lev Lewis, Wadood Y. Hamad, Mark J. MacLachlan

**Affiliations:** 1 Department of Chemistry, University of British Columbia, 2036 Main Mall, Vancouver, BC V6T 1Z1 Canada; 2 FPInnovations, 2665 East Mall, Vancouver, BC V6T 1Z4 Canada

## Abstract

Cellulose nanocrystals (CNCs) derived from biomass spontaneously organize into a helical arrangement, termed a chiral nematic structure. This structure mimics the organization of chitin found in the exoskeletons of arthropods, where it contributes to their remarkable mechanical strength. Here, we demonstrate a photonic sensory mechanism based on the reversible unwinding of chiral nematic CNCs embedded in an elastomer, leading the materials to display stimuli-responsive stretchable optics. Vivid interference colors appear as the film is stretched and disappear when the elastomer returns to its original shape. This reversible optical effect is caused by a mechanically-induced transition of the CNCs between a chiral nematic and pseudo-nematic arrangement.

## Abstract (web summary)

Cellulose nanocrystals (CNCs) spontaneously organize into a helical arrangement but flexible CNC elastomer composite materials with this structure were so far not realized. Here the authors demonstrate the fabrication of a CNC based elastomer composite and its application as a photonic strain sensor.

## Introduction

Over a span of more than 200 million years, arthropods such as crabs and lobsters have evolved some of the toughest known biocomposites in their exoskeletons^1^. At the heart of this composite, chitin is arranged into a helical assembly, called the Bouligand structure, with embedded protein and mineral (mostly calcium carbonate)^2^. This spiraling structure is effective to prevent crack propagation and sustain large impacts, which leads the exoskeletons to have impressive mechanical properties^3^. Interestingly, the same structure is responsible for the iridescence observed in some insects. When the distance of one rotation of the helical structure (pitch) is on the order of the wavelength of light, the structure can selectively diffract visible light^4^. In the jewel beetle, for example, a Bouligand structure of chitin nanocrystals is responsible for the iridescent elytra^5^. In fact, this property is not limited to chitin; cellulose in *Pollia condensata* also has a Bouligand-type structure that is responsible for the intense color of the fruit^6^.

Cellulose nanocrystals (CNCs) are spindle-shaped crystalline nanomaterials derived from biomass^7^. Owing to the unique features of CNCs that are not observed in bulk cellulose^8^, CNCs have been studied for potential applications in sensing^9^, tissue engineering^10^, reinforced plastics^11^, and optics^12,13^. The most notable property of CNCs is their ability to self-assemble into a chiral nematic organization that resembles the Bouligand structure^14–17^. In this organization, unidirectionally aligned CNC layers stack in a sequence where their orientation rotates with a periodic order. This helical structure obtained through evaporation-induced self-assembly (EISA) of CNCs is always left-handed^18–21^. When the pitch of the chiral nematic structure of CNCs is in the range of the wavelength of visible light, the thin films appear iridescent and selectively reflect left-handed circularly polarized light^22^.

Chiral nematic structures of CNCs have been transferred to several materials including silica^23^, organosilica^24^, and resins through EISA^25^; subsequent calcination or other treatment is used to remove CNCs. Although these materials are mesoporous, chiral, and display structural color, they are generally not very flexible and undergo plastic deformation or crack when flexed. So far, it has not been possible to make a highly flexible, homogeneous composite of chiral nematic CNCs embedded in an elastomer owing to incompatibility of hydrophilic CNCs and hydrophobic monomers or polymers, and the constraints of EISA to allow for CNCs to self-assemble. Moreover, most common elastomer precursors that can be polymerized by heat or photoinitiators (e.g., isoprene, butadiene, and acrylic esters) are highly volatile and would therefore evaporate during an EISA process that can take a few days at ambient temperature. Nonetheless, flexible films of CNCs have been formed by combining CNCs with polyethyleneglycol (PEG)^26^ or zwitterionic surfactants^13^, and by other approaches^27,28^.

In this study, we report a synthetic method that enables the construction of homogeneous, stretchable CNC/elastomer composites with a chiral nematic organization of CNCs. The chiral nematic CNC elastomer (CNC-E) is able to undergo large deformations by applying mechanical stress, and rapidly reverts to its original shape when the stress is removed. Most surprisingly, when the composite is stretched, the robust chiral nematic structure unwinds into a pseudo-nematic arrangement. Viewing the elastomer between crossed polarizers yields brilliant interference colors that dramatically change in response to extension and contraction.

## Results

### Chiral nematic organization of CNCs in an elastomer

**Fig. 1:**
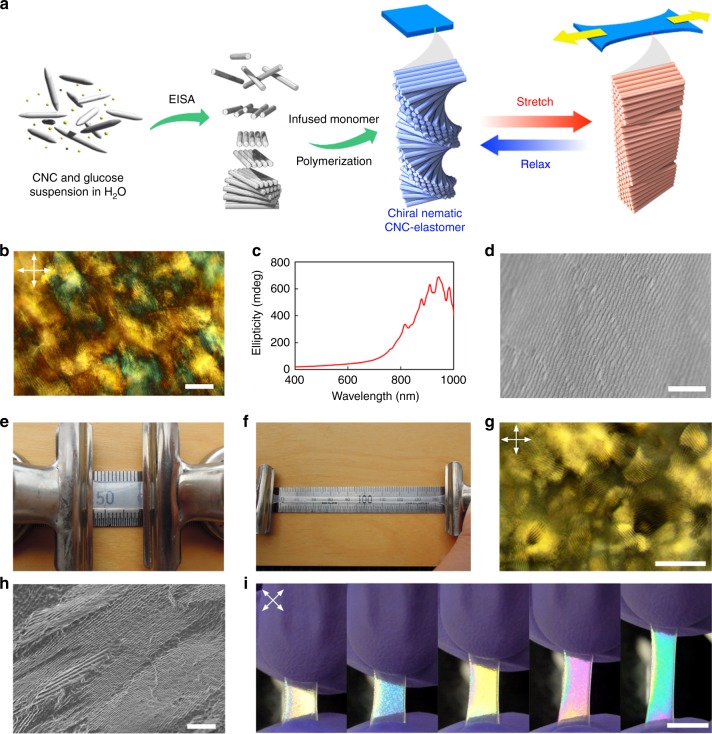
Preparation of chiral nematic cellulose nanocrystal-elastomer (CNC-E). **a** Schematic illustration of the preparation of the chiral nematic organization of CNCs and preparation process of CNC-E. **b** Fingerprint texture of chiral nematic structure in glucose-containing chiral nematic CNC (G-CNC) observed on film surface. Dark parts in fingerprints represent regions where incident linearly polarized light is converted to the other polarization states that do not transmit when the analyzer is arranged perpendicular to the polarizer. **c** Positive circular dichroism (CD) signal observed in G-CNC, confirming an overall left-handed chiral nematic arrangement. **d** Cross-section SEM image of G-CNC film. **e** Photos of unstretched and **f** stretched CNC-E. The stretching direction is perpendicular to helicoidal axis of chiral nematic structure of CNCs. **g** Fingerprint texture observed in an unstretched film of CNC-E when viewed by POM under crossed polarizers. **h** Cross-section SEM image of CNC-E film. **i** Photographs of CNC-E stretching as viewed under crossed polarizers. The color of the composite changes from white to blue to yellow to pink to green upon stretching. (Arrows in image indicate polarization axes of both linear polarizer and analyzer.) Scale bars represent **b** 50 µm, **d** 5 µm, **g** 100 µm, **h** 10 µm, and **i** 1 cm

The key challenge to preparing homogeneous composite films of CNCs with elastomers was to avoid phase separation of the hydrophobic monomers during EISA of CNCs. Our simple procedure overcomes this problem by improving the surface compatibility of the CNCs with an elastomer (Fig. 1a and Supplementary Figure 2). First, a film of CNCs and glucose was prepared by EISA from water. The obtained glucose-containing chiral nematic CNC (G-CNC) film appears iridescent, and fingerprint textures characteristic of chiral nematic order were found through polarized optical microscopy (POM) of the film (Fig. 1b and Supplementary Figure 3a). Strong positive signals were observed from circular dichroism (CD) experiments, confirming the presence of a left-handed chiral nematic organization of the CNCs in the film (Fig. 1c). In addition, scanning electron microscopy (SEM) of cross sections of the film revealed layered structures with periodic spacing and spiral stacking of CNCs that are characteristic of chiral nematic assemblies (Fig. 1d and Supplementary Figure 3b).

In the second step, the film was swollen with dimethyl sulfoxide (DMSO), then with elastomer precursors (ethyl acrylate: EA and 2-hydroxyethyl acrylate: 2-HEA in 8.4:1.0 mole ratio). This mixture of EA and 2-HEA was selected to obtain the optimal transparency and elasticity in the composite. Polymerization was initiated by 2,2′-azobis(2-methylpropionitrile) (AIBN) to give a homogeneous film of CNC/elastomer composite, CNC-E. In this method, glucose and DMSO play critical roles in allowing the CNCs to be compatible with the elastomer precursors. The addition of glucose minimizes cracking and inhomogeneities in CNC films, which would crack in the absence of glucose when drying. In addition, pure CNC films can be swelled with DMSO but the swelling is inhomogeneous. However, when glucose is added to CNC films, it acts as a polar additive to promote the uniform swelling of CNC films in DMSO. Films swollen with DMSO are then more amenable to diffusion and exchange with the elastomer precursors to produce homogeneous elastomeric films. This procedure was optimized after many failed attempts to create homogeneous films of chiral nematic CNCs in elastomeric matrices.

CNC-E is transparent and is able to stretch more than 900% when pulled (Fig. 1e, f, Supplementary Figure 12). POM observation of CNC-E also revealed a fingerprint texture, and cross-section SEM images showed the similar periodic structure as observed for G-CNC (Fig. 1g, h). The periodic spacings for G-CNC and CNC-E were 0.40 ± 0.12 and 0.69 ± 0.13 µm, respectively (error represents standard deviation, *n* = 20 and 35, respectively). As a control experiment, an elastomer with CNCs randomly arranged (see Methods section) did not show fingerprints by POM or periodic structures by SEM (Supplementary Figure 4). CNC-E preserves a chiral nematic arrangement of CNCs, but it does not show visible iridescence owing to the longer pitch in the structure as the polymer matrix was incorporated between the layers of CNCs.

**Fig. 2:**
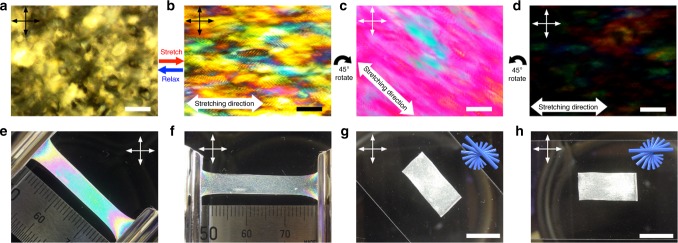
Polarized light observation of CNC-E. **a** Fingerprint texture of unstretched CNC-E as viewed by POM. **b** Distorted fingerprints observed in stretched CNC-E by POM under prolonged exposure time for photography. **c** Stretched CNC-E viewed between crossed polarizers by POM. Stretching direction was arranged 45° to polarizers. **d** POM image of stretched CNC-E under same exposure time for photograph as **c**. Stretching direction was arranged 0° to polarizer. **e**, **f** Stretched CNC-E film rotated between crossed polarizers. **g**, **h** Unstretched CNC-E film rotated between crossed polarizers. (Arrows in each image indicate polarization axes of both polarizer and analyzer.) Scale bars represent **a**–**d** 100 µm, and **g**, **h** 1 cm

Interestingly, when the sample was viewed between crossed polarizers, it showed vivid interference color changes as the sample was stretched (Fig. 1i). Supplementary Movie 1 shows the stretching of CNC-E viewed between crossed polarizers. Initially, CNC-E appears white, but as it is stretched, the observed interference color turns from white to blue, yellow, pink, then green; this sequence matches the order of colors in the Michel-Levy color chart as birefringence increases^29^. Interestingly, this phenomenon is reversible and the color disappears when the stress is removed and the composite rapidly returns to its original shape (Supplementary Movie 2). Moreover, POM characterization showed that the fingerprint texture of CNC-E is distorted during stretching (Fig. 2a, b). These results suggest that the color change arises from a change in birefringence due to a reorientation of CNCs in the composite. Although we anticipated a contribution from the EA/2-HEA copolymer matrix since aligned polymer chains have also been reported to show birefringence^30^, this was found to be negligible by conducting a control experiment with a sample that has both CNC-E and pure copolymer segments (Supplementary Figure 5). As the control sample is stretched, the CNC-E segments display the expected interference colors, but, in sharp contrast, the pure poly(EA/2-HEA) segment remains colorless when stretched and viewed under crossed polarizers. This outcome confirms the orientation of CNCs as the principal contributor to the color responsiveness of the CNC-E composite.

### Optical properties of relaxed and stretched CNC-E

There is a strong relationship between the observed color changes and the orientation of the composite relative to the polarizers. Vivid interference color was observed when the stretching direction was fixed at 45° relative to both polarizers, but the incident light was completely blocked when the stretching direction was parallel to the polarizer (Fig. 2c–f). Note Fig. 2b was exactly the same sample as 2d but was taken under prolonged exposure time for photography to allow us to take a brighter image showing distorted and conspicuous fingerprints that disappear in the 45° configuration. Indeed, rotating stretched CNC-E between the polarizers has a dramatic effect on the transmitted light intensity (Supplementary Figure 6 and Movie 3). Conversely, in the unstretched (static) CNC-E, the CNCs are arranged in a chiral nematic organization with the rods mostly situated parallel to the film. This arrangement leads to very little birefringence within the plane of the CNCs; there is no difference in transmitting light intensity and interference color when the film is rotated between the crossed polarizers (Fig. 2g, h and Supplementary Movie 4). These observations indicate that CNCs align in CNC-E when it is stretched, resulting in the observed birefringence.

**Fig. 3:**
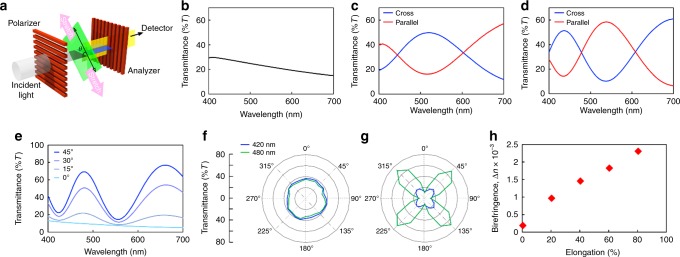
Polarized ultraviolet-visible (UV-vis) spectroscopy of CNC-E. **a** Schematic illustration of the experimental setup for measuring the transmission polarized UV-vis spectrum. **b** Transmitting polarized UV-vis spectra of static CNC-E between crossed polarizers. **c** Polarized transmission UV-vis spectra of 40%, **d** 80% elongated CNC-E under crossed and parallel arrangements of polarizers. **e** Polarized light intensities of stretched CNC-E. The sample was kept stretched and the stretching direction was oriented 0, 15, 30, and 45° to the polarization axis of the polarizer. **f** Polar plots of polarized light intensities of static and **g** stretched CNC-E relative to the polarizer. **h** Birefringence estimation of stretched CNC-E. Birefringence increases as the sample is stretched. (Stretching direction of samples are oriented 45° relative to the polarizer unless otherwise noted.)

Polarized ultraviolet-visible (UV-vis) spectroscopy measurements were conducted to further understand the optical behavior of stretched CNC-E. The orientations of the polarizers were fixed at 90°, and samples placed between the polarizer and analyzer were stretched along the direction that is 45° (*θ* = 45°) with respect to both polarizers (Fig. 3a). Static CNC-E transmitted the light without a particular signal in the range 400–700 nm, but light intensities gradually increased and peaks emerged as the sample was elongated (Fig. 3b–d). Moreover, the spectrum was inverted when the polarizer and analyzer were placed parallel to one another, resulting in the complementary color (Supplementary Figure 7 and Movie 5). When the elongated sample was kept stretched and rotated between crossed polarizers, the maximum transmittance was recorded when the stretching direction was 45° relative to both the polarizer and analyzer (Fig. 3e). The observation of constant intensities at all orientations for the static sample indicates that the CNCs in the unstretched composite are optically isotropic in the plane of the film (Fig. 3f). On the other hand, CNCs in the stretched composite are highly anisotropic and showed an optical axis aligned with the stretching direction (Fig. 3g).

In a typical birefringent material that has one optical axis, the transmitting polarized light intensities measured between crossed $\left( {I_ \bot } \right)$ and parallel $(I_\parallel )$ polarizers can be described by Eqs. (1)–(3) that depend on the orientation of the optical axis (i.e., the stretching direction of CNC-E in this study), where *d* is sample thickness, *λ* is wavelength, *R* is retardation, *θ* is angle, and Δ*n* is birefringence^31^.

1 $$I_ \bot = \sin 2\theta {\mathrm{sin}}^2\frac{{\pi d\Delta n}}{\lambda }$$

2 $$I_\parallel = \sin 2\theta {\mathrm{cos}}^2\frac{{\pi d\Delta n}}{\lambda }$$

3 $$\Delta n = \frac{R}{d}$$

The spectra from stretched CNC-E fit well to the theoretical spectra at a given film thickness (Supplementary Figure 8). Moreover, birefringence estimation obtained from polarized UV-vis spectroscopy revealed a gradual increase of birefringence as CNC-E was elongated (Fig. 3h). Based on these experimental results, we hypothesize that CNCs undergo a reversible transition from chiral nematic to pseudo-nematic orientation in the composite when the sample is stretched. In other words, the Bouligand structure of the CNCs unwinds during stretching and the CNCs become oriented parallel to the stretching direction of the elongated composite; they then return to their original chiral nematic structure when external stress is removed (see Supplementary Figure 12 for tensile testing).

### Reorientation of CNCs in stretched elastomer matrix

**Fig. 4:**
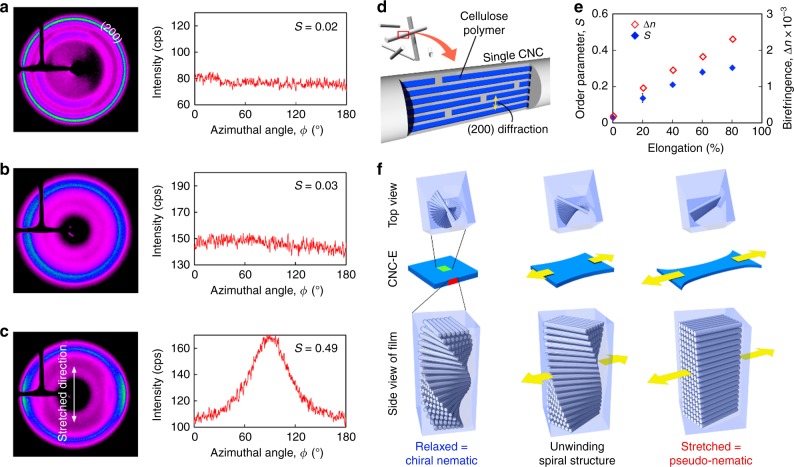
Two-dimensional X-ray diffraction analysis. **a** Diffraction pattern and intensity at 2*θ* = 22.9° of G-CNC, **b** unstretched CNC-E, and **c** stretched CNC-E (250% elongation). (The value in the plots is the calculated order parameter, *S*). **d** Schematic of idealized cross section of a single CNC nanocrystal, where the blue square represents a single cellulose polymer chain that is aligned with the CNC rod axis. X-ray diffraction of the (200) plane can be found at 2*θ* = 22.9°. **e** Plot of order parameter *S* and birefringence ∆*n* relative to elongation ratio. Error bars represent s.d. of two measurements. **f** Schematic illustration of reorientation of CNCs from chiral nematic to a pseudo-nematic structure as the sample is stretched

Orientation of CNCs under both relaxed and stretched conditions was evaluated by two-dimensional X-ray diffraction (2D-XRD). In the chiral nematic organization (unstretched state), CNC spindles are parallel to the film but point in every direction with equal probability. Thus, there should not be much angle dependence on the diffraction intensity. We looked at the intensity with respect to the azimuthal angle, $I(\phi )$, on the diffraction at 2*θ* = 22.9°, which corresponds to the (200) diffraction of cellulose I*β* crystal in CNCs^8^, when the X-ray beam is perpendicular to the plane of the film surface. As expected, both G-CNC and relaxed CNC-E, which have chiral nematic structures, show nearly homogenous diffraction patterns and intensities at 2*θ* = 22.9° (Fig. 4a, b). Stretched CNC-E, however, shows a strong angle dependence in its diffraction pattern and intensity as observed for a control sample of shear-aligned pseudo-nematic CNC film (Fig. 4c and Supplementary Figure 9). In addition, contributions from the polymer matrix at 2*θ* = 22.9° were found to be negligible (Supplementary Figure 10). To quantify the extent of CNC alignment, we calculated the Hermans order parameter (0 ≤ *S* ≤ 1, introduced by Eqs. (4)–(6)) based on the diffraction intensities at 2*θ* = 22.9° (Fig. 4d)^32^.

4 $$S = \frac{{3\left( {{\mathrm{cos}}^2\gamma } \right) - 1}}{2}$$

5 $${\mathrm{cos}}^2\gamma = 1 - 2\left( {{\mathrm{cos}}^2\phi } \right)$$

6 $${\mathrm{cos}}^2\phi = \frac{{\mathop {\int }\nolimits I\left( \phi \right){\mathrm{cos}}^2\phi \sin \phi\, \mathrm{d}\phi }}{{\mathop {\int }\nolimits I\left( \phi \right)\sin \phi\, \mathrm{d}\phi }}$$

We determined *S* = 0.02 and 0.03 (±0.01), respectively, for G-CNC and relaxed CNC-E, indicating a low degree of anisotropy in the plane of the film. In contrast, the order parameter increased as CNC-E was stretched (Supplementary Figure 11), reaching *S* = 0.49 at 250% elongation, which is close to that of our control shear-aligned pseudo-nematic CNC film. This agrees well with the observed interference color changes resulting from increased birefringence (Fig. 4e). These data support our claim that the orientation of the CNCs changes from chiral nematic order in the relaxed film to a partially aligned arrangement when stretched (Fig. 4f).

## Discussion

We demonstrated a highly stretchable, homogeneous elastomeric nanocomposite material containing chiral nematic structures of CNCs. The highly flexible composite can undergo over 900% elongation, returning to its original shape when external stress is removed (elongation without hysterisis observed within 300%). Remarkably, stretching of the polymer unwinds the chiral nematic organization of the CNCs, leading to nanocrystal alignment within the elastomer and, consequently, strong birefringence. The stretched composite shows vivid interference colors due to the emergence of an optical axis directed along the elongation direction. This surprising unwinding of the Bouligand structure is an interesting mechanism for developing reversible stimuli-responsive materials for applications in flexible optics and sensing, for instance. We further suggest that these materials may be useful for developing versatile sensors capable of visualizing mechanical stresses and simultaneously detecting the initiation of failure in various components, such as in bridges and buildings.

## Methods

### General

All reagents were purchased from standard suppliers and used without further purification. Aqueous suspensions of CNCs (2 wt%; pH 2.1) prepared from bleached kraft pulp were obtained from FPInnovations (Supplementary Figure 1). All materials for polymerization were purged with nitrogen before use. POM images were recorded on an Olympus BX41 microscope with linear polarizers. Transmission polarized UV-visible spectra were collected with an Agilent Cary 5000 UV-vis/NIR spectrometer. The sample was placed between linear polarizers, which were oriented parallel or crossed as indicated in the text and figure captions. CD spectroscopy was performed in transmission mode using a JASCO J-710 spectrometer. SEM was performed using a Hitachi S4700 electron microscope on gold sputter-coated samples. 2D-XRD images were recorded with a Bruker APEX DUO with APEX II CCD Detector using Cu Kα_1_ X-ray beam with a wavelength (*λ*) of 0.154 nm at 0.6 mA, 45 kV for 480 s at 60 mm from the detector in transmission mode. Tensile testing was performed on an Instron 5566Q at a rate of 50 mm min^−1^.

### Preparation of G-CNC

*α*-d-glucose (50 mg) was added to an aqueous CNC suspension (2.0 wt%, pH 2.1, 5.0 g), the mixture was sonicated for 15 min, and then was poured into a polystyrene dish (60 mm diameter). EISA was carried out at ambient temperature for 3 days to obtain an iridescent G-CNC film with thickness of approximately 70 µm.

### Preparation of chiral nematic CNC-E

A square piece (15 × 15 mm) of G-CNC film was placed in a vial and purged with nitrogen for 8 min. The film was soaked in an AIBN solution (0.15 M in DMSO) for 8 min to swell the film under nitrogen. Excess DMSO was removed with a syringe. A mixture of EA (1.2 mL), 2-HEA (0.15 mL), and AIBN (1.81 mg) was added. The sample was kept still for 4 h to enable the elastomeric monomers to permeate the DMSO-swollen G-CNC film. Finally, a glass slide was placed on the monomer-infused film and purged with nitrogen for 8 min. Polymerization was initiated by heating the cell to 60 °C over 1 h and the cell was held at 60 °C for 18 h to capture the chiral nematic organization of CNCs in an elastomer. The obtained CNC-E contains approximately 9 wt% of CNCs and is 600 (±10) µm in thickness. Other elastomeric precursors (e.g. isoprene, polydimethylsiloxanes, and other acrylic esters) and radical initiators can be applied to manipulate mechanical properties of the composite; however, we found EA/2-HEA copolymer was most appropriate in terms of transparency.

### Preparation of shear force-aligned pseudo-nematic CNC film

An aqueous suspension of CNCs was concentrated to 11 wt% by evaporation of water. Concentrated CNC suspension (11 wt%, pH = 2.1, 200 mg) was cast between two rectangular spacers (50–400 µm thickness) aligned in parallel that were placed on a glass slide. The CNC suspension was then covered by another glass slide and the suspension was sheared by moving the cover slide back and forth over the suspension at a rate of approximately 2 cm s^−1^ for 40 times. After removal of top glass slide, the sheared CNC suspension was dried at ambient temperature overnight to obtain the pseudo-nematic CNC film.

### Preparation of a random CNC film

To an aqueous suspension of CNCs (pH 2.1, 2.0 wt%, 5 g), 2.0 wt% of NaCl (2 mg) was added and sonicated for 15 min, then the mixture was poured into a polystyrene dish (60 mm diameter) for drying. A non-iridescent and transparent free-standing film was obtained with thickness of approximately 70 µm.

### Preparation of random CNC-E composite

A square piece (10 × 10 mm or 5 × 10 mm) of random CNC film was cut out then soaked with an AIBN solution (0.15 M in DMSO) in a nitrogen-purged cell for 30 min to swell the film. Excess DMSO was removed then a mixture of EA (1.2 mL), 2-HEA (0.15 mL), and AIBN (1.81 mg) was added. The sample was kept still for 4 h to enable the elastomeric monomer to permeate the DMSO-swollen random CNC film. Finally, a glass slide was placed on the monomer-infused film then polymerization was initiated by heating the cell to 60 °C over 1 h and the cell was held at 60 °C for 18 h to capture the random organization of CNCs in an elastomer.

### Preparation of CNC-E containing acrylate copolymer

A square piece (5 × 10 mm) of G-CNC film was cut out and placed on a 10 × 10 mm glass slide where G-CNC is aligned along one edge of the slide. The film was then soaked with an AIBN solution (0.15 M in DMSO) in a nitrogen-purged cell for 30 min to swell the film. Residual DMSO was removed then a mixture of EA (1.2 mL), 2-HEA (0.15 mL), and AIBN (1.81 mg) was added. The monomer mixture also covered the half of the glass slide where no G-CNC was placed; this is supposed to become the pure copolymer matrix. The sample was kept still for 4 h to enable the monomer to permeate the DMSO-swollen G-CNC film. Finally, a glass slide was placed on top of the monomer-infused film, then polymerization was initiated by heating the cell to 60 °C over 1 h and the cell was held at 60 °C for 18 h to capture a polymer film with two segments: half CNC-E and half pure polymer matrix.

### Polarized UV-vis spectroscopy

Samples are placed between polarizers that are arranged in crossed or parallel configurations. In case of stretched samples, samples were kept elongated and the stretching direction was fixed at 45° relative to the polarization axis of the polarizers. Sample dimensions (thickness, length of original and stretched) were measured beforehand.

### Estimation of birefringence in stretched CNC-E

There is some literature describing birefringence determination of nematic (anisotropic) CNCs on the basis that they are regarded as one-optical-axis birefringent materials^31,33^. However, anisotropic characteristics of CNC-E in this study vary depending on the extent of elongation, and it is relatively less anisotropic in small elongation. Thus, the reported methods are not very applicable to our study. To address this issue, we applied another method to estimate birefringence of stretched CNC-E. Birefringence of CNC-E in static condition (i.e., before stretching) was estimated based on Michel-Levy color chart because CNCs in that condition are optically isotropic in macroscopic scale due to homogenous alignment of CNCs within the film plane; they do not follow the behavior of a one-optical-axis birefringent material. Transmitting polarized UV-vis spectra of uniaxial birefringent materials between crossed and parallel configurations of polarizers can be described by Eqs. (1)–(3), where *R* is retardation (nm), *d* is sample thickness (nm), ∆*n* is birefringence, and *I*_⊥_ and $I_\parallel$ are transmitting intensities of incident light *I*_0_ under crossed and parallel configurations of polarizers, respectively^31^. When the stretching direction of the sample was fixed at 45° (*θ* = 45) relative to the polarization axis of the polarizers, absorbance and transmittance contrast can be determined for thickness *d* and retardation *R* in the above equations. With measured sample thickness, theoretical spectra can be fit to experimental results by means of nonlinear least-square method seeking optimal value for *R*; birefringence ∆*n* was calculated from the obtained *R*. Note this method does not incorporate wavelength-dependent dispersion of birefringence.

### Calculation of Hermans order parameter *S*

Diffraction intensities *I*(*ϕ*) with respect to the azimuthal angle *ϕ* were recorded at 2*θ* = 22.9°, which corresponds to the (200) diffraction of cellulose I*β* crystal oriented along the rod axis of CNC. The plots were then fit to the combination of two equal Lorentz distributions by the least-squares fitting method to obtain the profile of *I*(*ϕ*). The order parameter *S* was calculated from *I*(*ϕ*) following Eqs. (4)–(6)^32^. Here *ϕ* represents the directional angle of the (200) reflection in CNC-E relative to the stretching direction (which corresponds to *ϕ* = 0° in this measurement).

### Measurement of tensile strength

Portrait-shaped specimens (7 × 15 mm) were cut out from one piece of CNC-E film. Elastic modulus was calculated from measured stress vs. strain curves and cross-section area of each sample.

## Supplementary information

Supplementary Information

Description of Additional Supplementary Files

Supplementary Movie 1

Supplementary Movie 2

Supplementary Movie 3

Supplementary Movie 4

Supplementary Movie 5

## Data Availability

The data that support the findings of this study are available within the article and its Supplementary Information File or from the corresponding author upon reasonable request.
