# Supplementary material for: Unwinding a spiral of cellulose nanocrystals for stimuli-responsive stretchable optics
Source: Nat Commun. 2019 Jan 31;10:510. doi: 10.1038/s41467-019-08351-6 (PMC6355765; doi:10.1038/s41467-019-08351-6)
Supplement: Supplementary file 1 — Supplementary Information [file 41467_2019_8351_MOESM1_ESM.pdf]

Supplementary Information for

Unwinding a Spiral of Cellulose Nanocrystals for Stimuli-Responsive Stretchable Optics

*Osamu Kose, Andy Tran, Lev Lewis, Wadood Y. Hamad, Mark J. MacLachlan**

List of Supplementary Information

Supplementary Figures 1-12

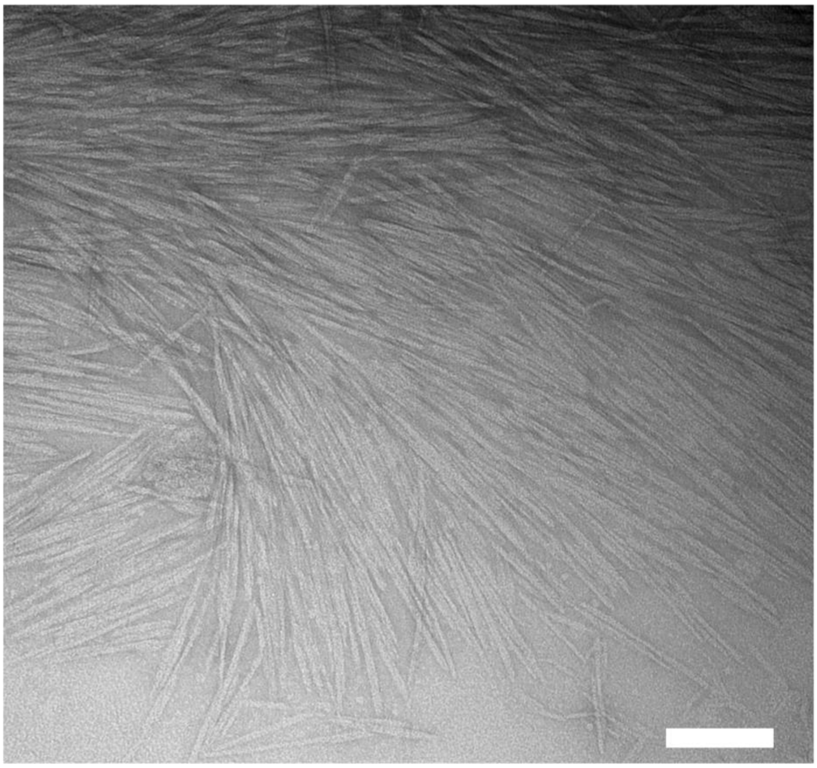

Supplementary Figure 1. Transmission electron microscopy (TEM) image of CNCs. Scale bar represents 100 nm.

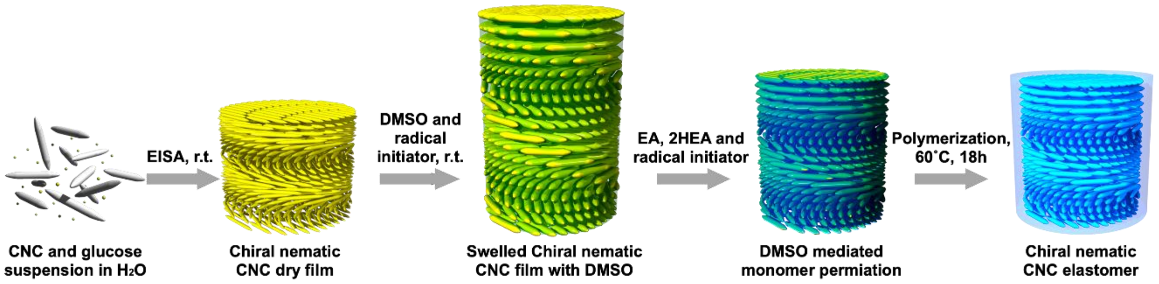

Supplementary Figure 2. Schematic illustration of **CNC-E** preparation. An aqueous suspension of CNCs and glucose cast on a polystyrene petri dish underwent EISA to form chiral nematic CNC dry film. The dried film was dipped into a DMSO solution containing radical initiator to swell followed by addition of monomers and radical initiator. Polymerization was started by heat to capture the chiral nematic structure of CNCs in the elastomer.

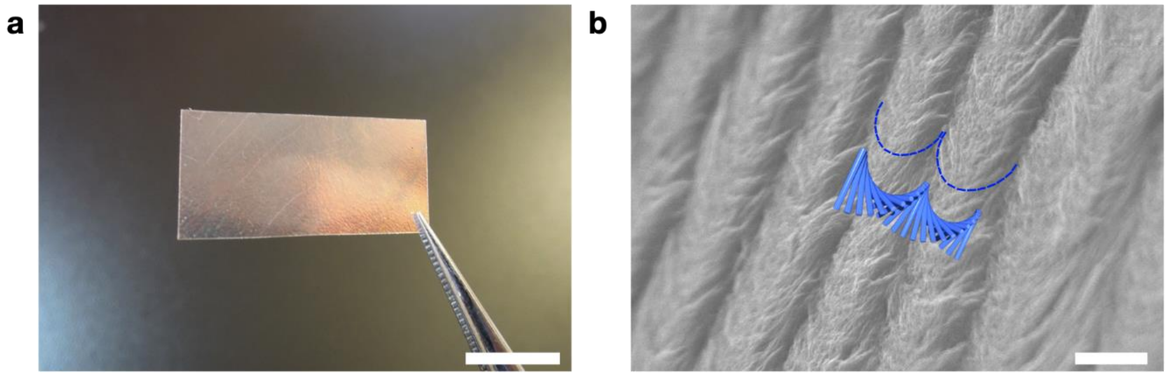

Supplementary Figure 3 a Photograph of a free-standing dried film of **G-CNC** viewed under normal white light reveals iridescence. **b** Cross section images of **G-CNC.** Layered structures were observed throughout cross sections of the film. Stacking of unidirectionally aligned CNCs layers in left-handed manner. Scale bars represent (a) 1 cm, and (b) 500 nm.

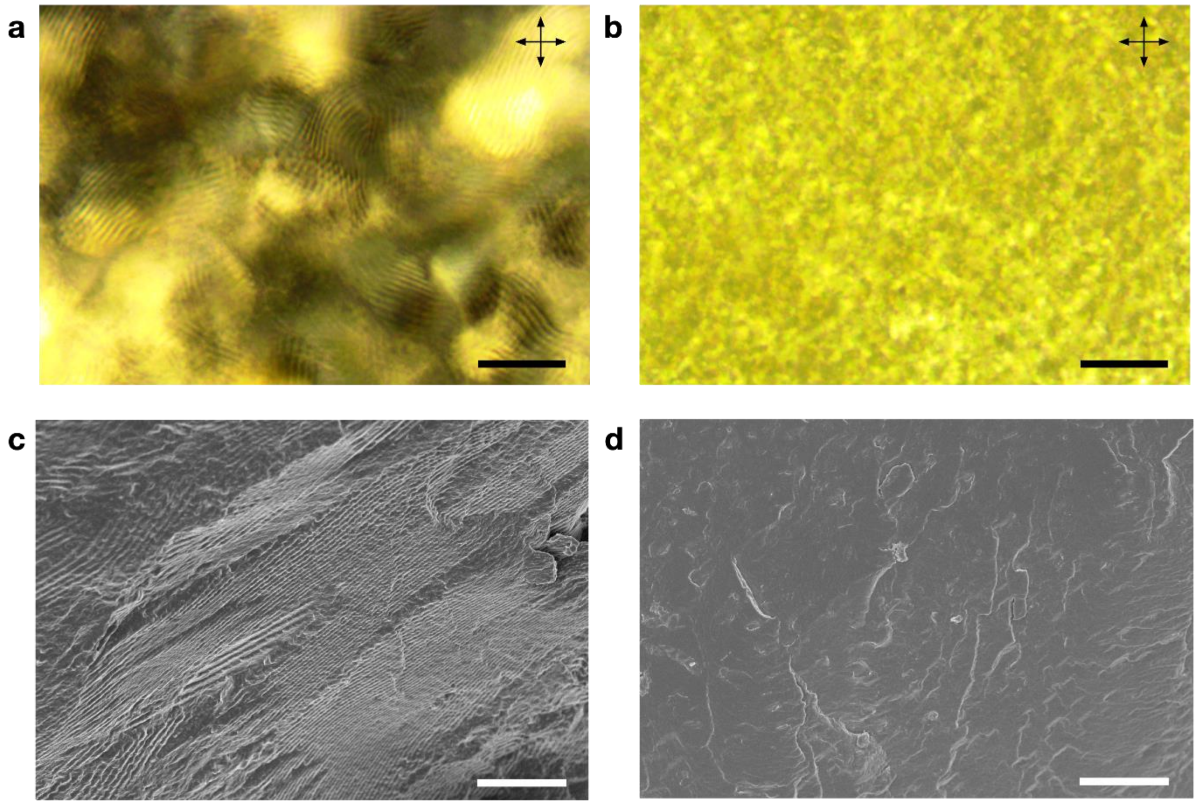

Supplementary Figure 4 a Fingerprint texture of unstretched **CNC-E** observed by POM. **b** Random CNC-elastomer film observed by POM. No evidence for a fingerprint texture was found across the surface of the film. **c-d** Cross section SEM images of static **CNC-E** and random CNC-elastomer. While **CNC-E** clearly shows a periodic, layered structure throughout the cross section, there are no features for this structure in the random CNC-elastomer. (Arrows in the images indicate orientation of the polarization axes of both linear polarizer and analyzer.) Scale bars represent (a-b) 50 µm, and (c-d) 20 µm.

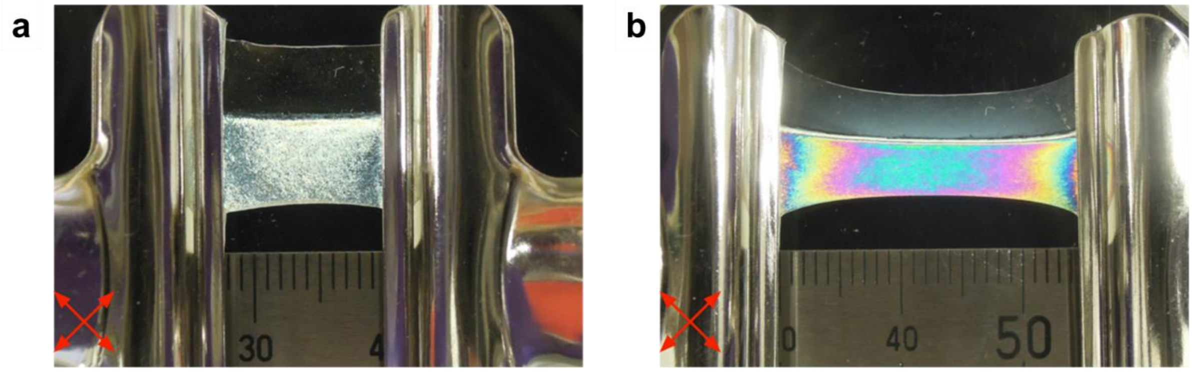

Supplementary Figure 5. Control experiment to compare the optical properties of **CNC-E** and pure copolymer matrix. **a** **CNC-E** having a pure copolymer segment (top) in static condition (unstretched). Because the pure copolymer segment in relaxed condition has no birefringence, incident linear polarized light cannot transmit through the analyzer arranged in cross configuration. Chiral nematic CNCs in static elastomer show a small birefringence, giving the white color. **b** **CNC-E** and pure copolymer in stretched condition. Due to aligned polymer chains in the pure copolymer, it showed birefringence and slightly transmitted incident light, but the birefringence change is very small compared to that for **CNC-E**. These outcomes imply that the birefringence change arising from mechanical stress originates predominantly from the reorientation of the CNCs rather than from alignment of the copolymer matrix.

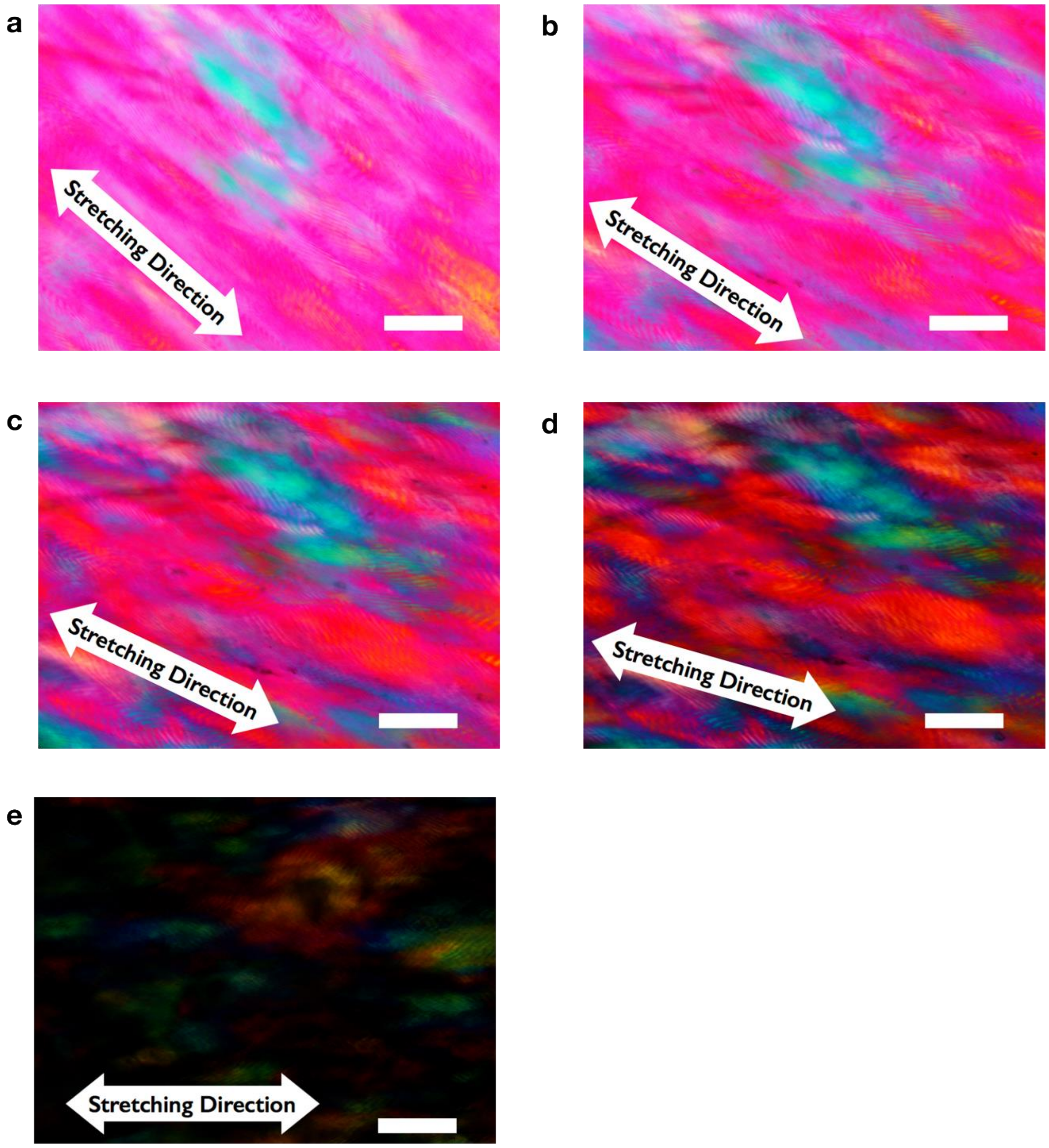

Supplementary Figure 6. POM observation of stretched **CNC-E**. **a-e CNC-E** was stretched and held still, then the stretching direction was changed from 45° to 0° relative to the polarization axis of the polarizer. As the angle neared 0°, transmitting light intensities decreased and dark parts of fingerprints appeared. Scale bars in figures indicate 100 µm, and they are parallel to the polarization axis of the polarizer. (Analyzer was arranged as crossed and light exposure time for photograph was 12 ms for all conditions.)

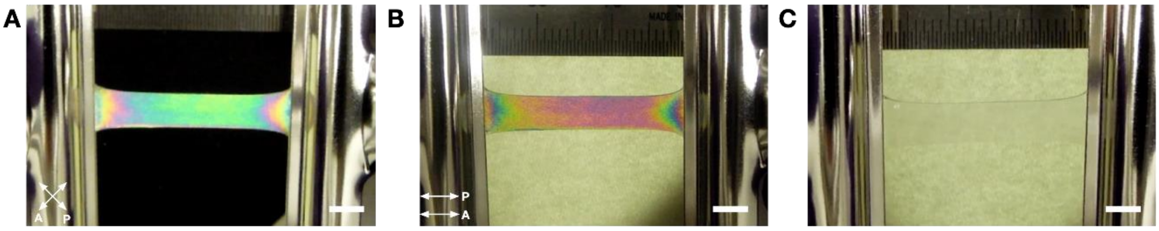

Supplementary Figure 7. Complementary color of stretched **CNC-E**. **a** Polarized light observation of stretched **CNC-E** with crossed polarizer / analyzer and **b** parallel configuration of polarizer / analyzer. The interference color in both configurations is complementary, behavior consistent with a uniaxial optically birefringent material. **c** Stretched **CNC-E** viewed without polarizers. Scale bars represent 5 mm.

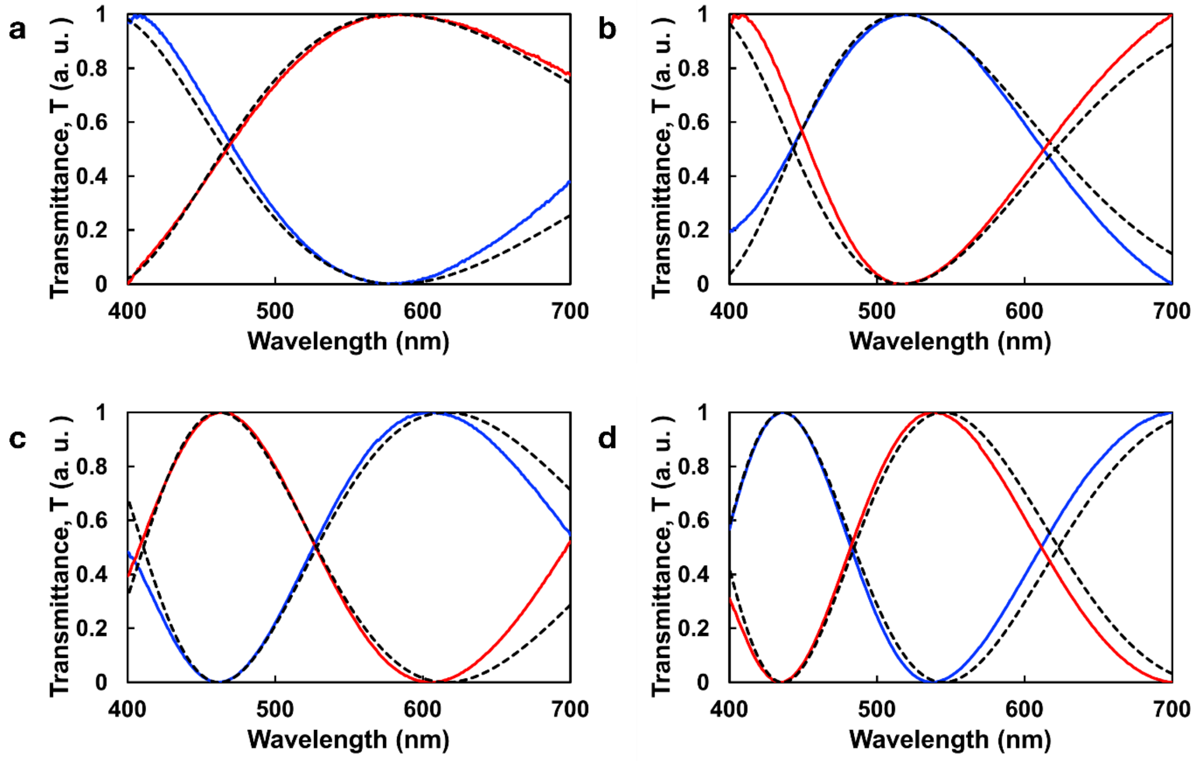

Supplementary Figure 8. Theoretical fitting of experimentally recorded polarized UV-vis spectra. **a** Experimentally obtained spectra (solid line) in both cross and parallel configuration of stretched **CNC-E** at 20%, **b** 40%, **c** 60%, and **d** 80% elongation. The fitted theoretical spectra (dotted lines) were derived from the aforementioned method. Transmission spectra are in good agreement with theoretical spectra describing a uniaxial optically birefringent material. Good fitting at 20% elongation implies CNCs in 20% stretching composite are already mostly aligned into the pseudo-nematic structure.

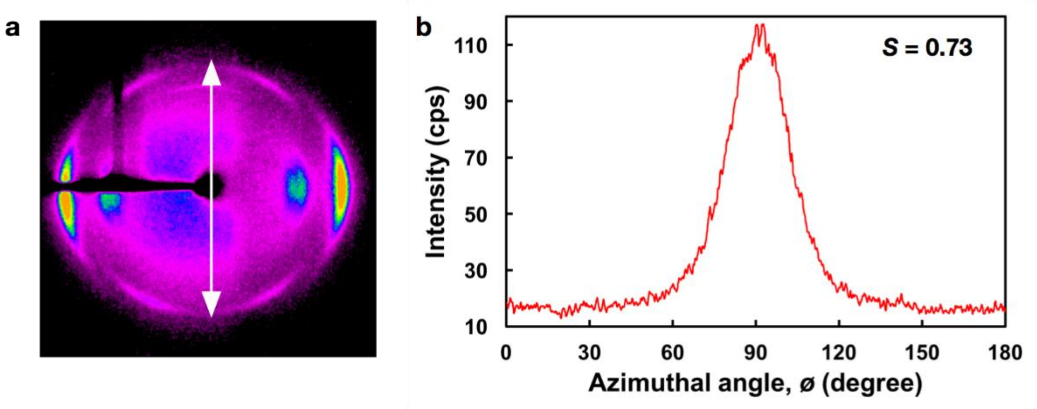

Supplementary Figure 9. 2D X-ray diffraction analysis of pseudo-nematic CNC film. **a** Diffraction pattern of pseudo-nematic CNC film where CNCs are shear-aligned along with vertical direction in image. Diffraction at $2\theta = 22.9°$ representing the $d(200)$ plane of the cellulose polymer chain in each CNCs oriented 90° relative to align direction. This image resembles that of stretched **CNC-E**. **b** Diffraction intensity at $2\theta = 22.9°$ toward azimuthal angles and calculated order parameter $S = 0.73$. (An arrow in the figure indicates the alignment direction of CNCs.)

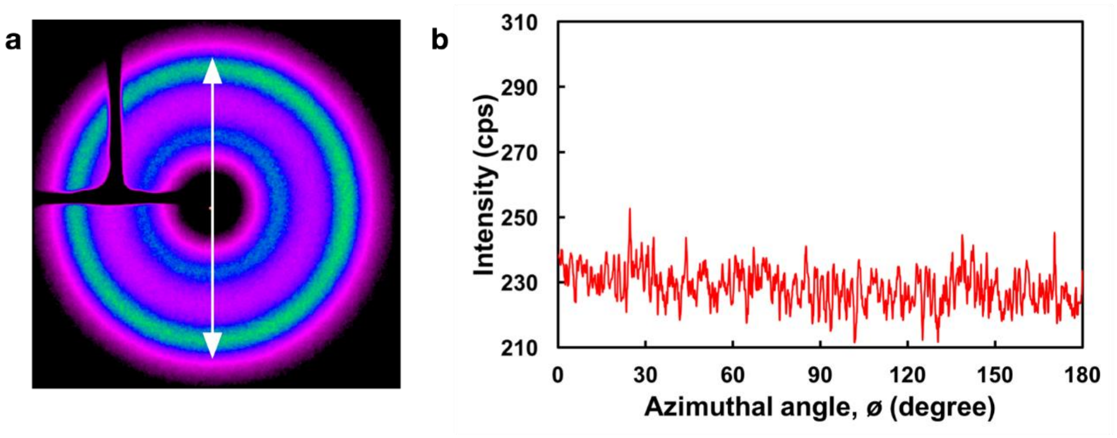

Supplementary Figure 10. 2D X-ray diffraction analysis of stretched pure copolymer (ethyl acrylate/2-hydroxyethyl acrylate) film (no CNCs). **a** Diffraction pattern of stretched (250% elongation) pure copolymer. **b** Diffraction intensity at 2θ = 22.9°. (An arrow in the figure indicates the stretching direction.)

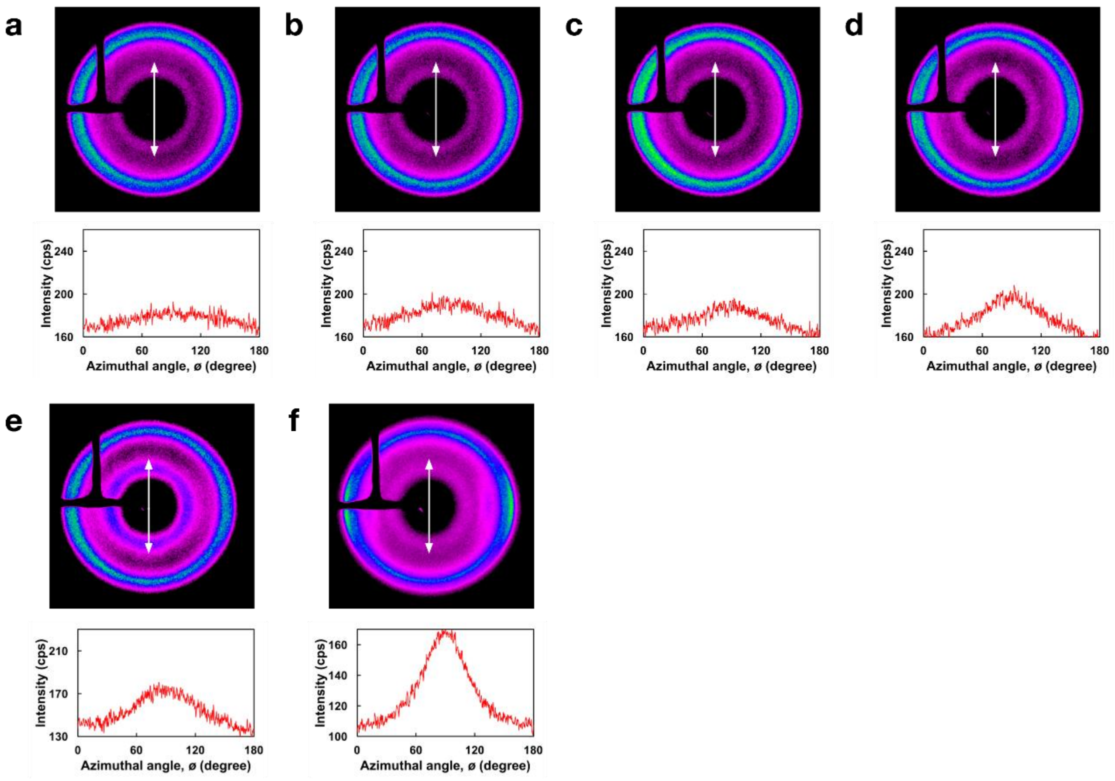

Supplementary Figure 11. 2D X-ray diffraction analysis of stretched **CNC-E**. **a** Diffraction pattern and intensity at 2θ = 22.9° of stretched **CNC-E** at 20%, **b** 40%, **c** 60%, **d** 80%, **e** 150%, and **f** 250% elongation. (An arrow in the figure indicates the stretching direction of the film.)

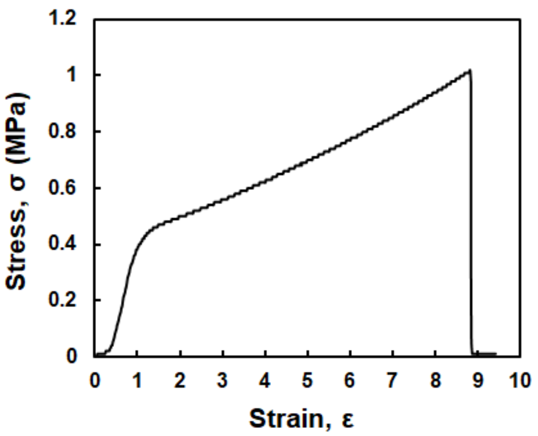

Supplementary Figure 12. Representative stress-strain curve of **CNC-E**. Tensile modulus of **CNC-E** was measured to be 1.1 ± 0.4 MPa with a tensile strain of 9.2 ± 1.4 (error represents standard deviation, n = 4).
