# Supplementary material for: Unwinding a spiral of cellulose nanocrystals for stimuli-responsive stretchable optics
Source: Nat Commun. 2019 Jan 31;10:510. doi: 10.1038/s41467-019-08351-6 (PMC6355765; doi:10.1038/s41467-019-08351-6)
Supplement: Supplementary file 2 — Description of Additional Supplementary Files [file 41467_2019_8351_MOESM2_ESM.pdf]

Description of Additional Supplementary Files

Supplementary Movie 1

Interference color change of CNC-E. Reversible optical property of CNC-E as stress was applied and removed. Polarizers were arranged perpendicular to one another (crossed) and CNC-E was elongated 45° relative to the polarization axis of the polarizer.

Supplementary Movie 2

Quick response of CNC-E to rapid stress application and removal. CNC-E showed rapid and reversible interference color changes when a stress was applied. Polarizers were arranged perpendicular to one another (crossed) and CNC-E was elongated 45° relative to the polarization axis of the polarizer.

Supplementary Movie 3

Extinction and diagonal position of stretched CNC-E. Polarizers were arranged perpendicular to one another (crossed) and CNC-E was stretched along the polarization axis to the extinction position, then rotated 45° to the diagonal position where maximum light transmittance was obtained.

Supplementary Movie 4

Polarized observation of static CNC-E rotating against polarization axis of polarizer. Polarizer and analyzer were arranged perpendicular to one another (crossed) and static CNC-E was rotated.

Supplementary Movie 5

Complementary color of stretched CNC-E viewed under parallel configuration of polarizers. With polarizer and analyzer aligned parallel to one another, stretched CNC-E shows complementary color to that observed when the polarizer and analyzer are perpendicular (crossed) to one another.
